# Antidiarrheal activity and acute oral toxicity of *Mentha longifolia *L. essential oil

**Published:** 2015

**Authors:** Ghader Jalilzadeh-Amin, Massoud Maham

**Affiliations:** 1 *Department of Clinical Sciences, Faculty of Veterinary Medicine, Urmia University, Urmia, Iran*

**Keywords:** *Intestinal transit*, *Diarrhea*, *Antispasmodic*, *Essential oil*, *Castor oil*, *LD50*

## Abstract

**Objectives:**

*Mentha longifolia* L. (*Lamiaceae*) is an annual herb that is used in the Iranian traditional medicine for treating stomach and intestinal disorders. The purpose of this study was to determine the protective effect of *M. longifolia* on experimental diarrhea in a rat model.

**Materials and Methods:**

The antidiarrheal activity of essential oil of *M. longifolia* (20-80 mg/kg) was investigated against castor oil-induced diarrhea in rats using loperamide as the standard reference drug. In acute toxicity evaluation, rats were orally administrated with single dose of EOML at doses ranging from 10 to 1000 mg/kg.

**Results:**

EOML caused a significant (*p*<0.05) and dose-dependent decrease of gastrointestinal transit, nevertheless, it could not block the inhibitory effect of atropine (0.1 mg/kg). EOML at oral doses of 20 and 80 mg/kg protected the animals against castor oil-induced diarrhea significantly (*p*<0.05). EOML decreased the intestinal fluid accumulation as indicated by the significantly (*p*<0.05 to *p*<0.001) decrease compared to control. The oral LD_50_ of EOML was found to be 470 mg/kg in rat.

**Conclusion:**

Since the inhibition of intestinal hyperactivity and hypersecretory are the bases of the treatment of diarrhea, results obtained in the present study suggest that EOML is endowed with antidiarrheal activity. EOML is moderately toxic for oral medication.

## Introduction

Diarrheal diseases are a major health concern and major causes of mortality in developing countries with an estimated 1.5–2 million deaths among children every year (Ouyan and Chen, 2004 ▶). Despite improvements in public health, it is a significant clinical problem in developed countries and estimated that will remain as a global health concern in the future (Meyrowitsch and Bygbjerg, 2007 ▶). Diarrhea is the passage of liquid stools and more frequent than normal defecation per day. Generally, it is an indication of gastrointestinal disorder, which can be caused by a variety of mechanisms. Principals of diarrhea treatment are rehydration therapy, antibiotics, and gut motility suppressing agents, which aim to reverse dehydration, reduce the severity of the illness, and reduce the period of convalescence time (Palombo, 2006 ▶). Nowadays, among the infectious diarrhea, some of inducement enteropathogens have a high resistance to commonly used antibiotics and even resist quickly to new drugs. Diarrheal diseases are more common and may be life-threatening, particularly when children and people who are malnourished or have impaired immunity are involved (Gilani et al., 2005 ▶). Therefore, search for new drugs with antidiarrheal properties is a public health priority. The ethno-botanical studies and previously reported antidiarrheal activity of many medicinal plants suggest that these might be an alternative in the treatment of diarrhea (Martini and Eloff, 1998 ▶; Rates, 2001 ▶).

*Mentha* sp. belongs to *Lamiaceae* family and comprises approximately 25-30 species which are found in temperate regions of world. Plants of this genus are perennial, herbaceous, and characterized by their aromatic volatile oils which are of great economic importance, being used by the flavor and fragrance in food preparation and pharmaceutical industries (Damien et al., 2003; Zargari, 1990 ▶). *Mentha longifolia* a member of this genus is widely cultivated for production of oils or leaves in Iran. It is a well-known remedy in Iranian traditional medicine for treatment of various ailments particularly for its valuable effects on the alimentary tract functions. It has been used as a folk remedy because of antiemetic, diaphoretic, antispasmodic, analgesic, stimulant, emmenagogue, and anticatharrhal activities. Moreover, it has been used in treating bronchitis, flatulence, anorexia, ulcerative colitis and stomach, and intestinal disorders such as cramps, nausea, indigestion, and diarrhea (Iscan et al., 2002; Moreno et al., 2002; Zargari, 1990 ▶). Antibacterial (Mimica-Dukic et al., 2003), antimycotic (Abou-Jawad et al., 2002), anthelmintic (Kozan et al., 2006), antioxidant (Mimica-Dukic et al., 1999), and antispasmodic (Jalilzadeh-Amin et al., 2012 ▶; Shah et al., 2010 ▶) activities have been confirmed for *M. longifolia*. 

The main constituents of the essential oil obtained from leaves of M. longifolia include Pulegone (15.55%), 1,8-cineole (18.93%), caryophyllene oxide (14.8%), and isomenthone (12.4%). Others minor components include sabinene, pinenes, isopulegone, piperitone, caryophylene oxide, neryl acetate, and geranyl isovalerate (jalilzadeh-Amin et al., 2011 ▶). In recent years, medicinal plants have gained popularity as prospective antidiarrheal agents, with a large numbers of studies being published in the past decade (Gutierrez et al., 2007 ▶). In view of this, the current study aimed to evaluate the antidiarrheal activity of essential oil of *M. longifolia* in castor oil-induced diarrhea in rats.

## Materials and Methods

**Animals**

Wistar rats (200 - 230 g) obtained from the Central Animal House of Urmia University were used after 2 week of acclimation. The animals were housed under standard environmental conditions at room temperature (23±2 °C), humidity 55–60%, and 12 h light/dark cycle and fed with a standard diet (Dane-Pars Co. Iran) and tap water *ad libitum*. Food was withheld 18-24 h prior to experimentation but they were allowed free access to water. All the experiments reported in this study were carried out in accordance with current guidelines for the care and use of laboratory animals (NIH guidelines).

**Preparation of essential oil and chemical agents**

*Mentha longifolia* were collected near the city of Urmia, Iran, in August 2013. It was identified by Dr Shahrokh Kazempour, from the Department of Plant Biology of the Tarbiat Modares University, Tehran, Iran. A voucher specimen (number 3571-B) of this plant was deposited at the herbarium of the Institute. Essential oil of *M. longifolia* (EOML) was extracted from fresh aerial parts of plants by hydrodistillation for 3 h. The essential oil was kept protected from light at 4 °C. Castor oil (a laxative agent), Tween 80, and charcoal meal (10% activated charcoal in 5% tragacanth powder) were purchased from Merck Co., Germany. Loperamide and atropine sulphate, were obtained from Sigma Chemical Co., USA. To perform the experiments, EOML was dissolved in 2% Tween. Charcoal meal, atropine sulphate, and loperamide were dissolved in normal saline.

**Acute oral toxicity study**

The acute oral toxicity of EOML was measured in accordance with Lork’s method (Lork, 1983). Following 18 hr fasting, animals were randomly divided into eight groups. Animals in the group 1 were considered as the control received essential oil vehicle and were administered 2 ml of Tween 80 (2%). Groups 2 to 8 received the following doses of EOML separately: 10, 100, 140, 225, 370, 600, and 1000 mg/kg diluted in Tween 80 (2%). All of the treatments were administered as a single oral dose through an oral intubation cannula. Pelleted diet was made available 2 hr after dosing, and free access to the water was presented. The general behaviors of the rats were continuously monitored for 1 hr after dosing, periodically during the first 24 hr, and then daily thereafter, for a total of 14 days. The LD_50_ was then calculated based on the mortality using the Probit-log analysis. 

**Intestinal transit time**

The effect of EOML on intestinal transit time (ITT) was measured after a charcoal meal using the method described by Aye-Than et al. (1989). Briefly, the fasted rats for 18 hr were randomly divided into 7 groups (six animals for each group). Animals in groups 1-4 received 80, 60, 40, and 20 mg/kg of EOML, respectively. The animals in group 5 received equal volume of the vehicle alone, while those in group 6 were given atropine (a standard antispasmodic agent) at a dose of 0.1 mg/kg. The seventh group received atropine sulphate 30 min before EOML (60 mg/kg) administration. Half an hour later each animal was treated orally with charcoal meal as a marker diet. Thirty minutes after the charcoal meal was given, all of the animals were euthanized by cervical dislocation, the abdomens were cut open, and the intestines were carefully removed from cardia to the anus. The intestines were immediately immersed in formalin to arrest peristalsis.

Thereafter, the distance the meal traveled through the small intestine was measured in every individual rat and reported as peristaltic index (PI). The inhibition of gastrointestinal transit of the charcoal meal by atropine and EOML was calculated as a function of the negative control using the following formula:

Percentage inhibition =N – n/N × 100

In this formula, "N" represents the length traveled by charcoal meal in the non-treated rats and "n" represents the length traveled by the charcoal meal in the treated animals.

**Castor oil-induced diarrhea in rats**

Following the method described by Atta and Mouneir (2005) 18-h fasted rats of either sex weighing 200-220 g were divided into four groups (6 animals in each). Group 1 served as negative control (10 mL/kg of 2% Tween 80 solution, p.o.), groups 2 and 3 received 80 and 20 mg/kg (p.o.) of EOML while group 4 as the positive control received loperamide (3 mg/kg, p.o.). One hour after treatment, diarrhea was induced by oral administration of 2 ml castor oil in each animal. Afterwards, they were placed separately over clean filter papers inside cages which were replaced every hour.

 The filter papers were inspected for the presence of diarrheal droppings at hourly intervals for a period of 4 hr. The following parameters were recorded: the onset of diarrhea, the total number of fecal outputs, and the total weight of diarrheic stools excreted for each animal in 4 hr. The severity of the castor oil-induced diarrhea was noted and recorded as a score. A numerical score based on stool consistency was assigned as follows: normal stool (or absence of diarrhea), semi-solid stool, and watery stool/feces. . The results were detailed by taking the vehicle groups as 100% and calculated as percentage inhibition for the treatment and positive control groups.

**Castor oil-induced intestinal transit in rats**

As described above, the rats used were fasted for 18 hr (the animals had free access to tap water) and randomly allocated into four groups of six rats per group. The animals in all groups were treated orally with EOML (80 and 20 mg/kg), loperamide (3 mg/kg), and with the vehicle (10 mL/kg) 30 min before the administration of castor oil (2 ml/rat). Then, 30 min following oral treatments of all of the animals with castor oil, each animal in the four groups were given 2 mL/kg (p.o.) of standard charcoal meal (10% suspension in 5% tragacanth powder). All of the animals in each treatment group were sacrificed 20 min after administration of the charcoal meal and their small intestines were immediately isolated. The traveled distance of the charcoal plug from pylorus to caecum, as the peristaltic index (PI) was determined and expressed as a percentage of the total length of the small intestine (Aye-Than et al., 1989).

**Castor oil -induced fluid accumulation**

The experiments were performed as described by Robert et al. (1976) with some modifications. Briefly, Wistar rats (150–200 g) fasted for 18 hr were allotted into 4 groups with six animals in each group. The animals were pretreated orally with loperamide (3 ml/kg), EOML (80 and 20 mg/kg), and Tween 80 (10 ml/kg). One hour later, the animals were administered with castor oil (2 mL/rat), intragastrically. The rats were sacrificed by cervical dislocation 1 hr later and their small intestines were removed after ligation at the pyloric end and the ileocaecal junction, respectively. The intestinal contents were then expelled into a graduated tube and its volume was measured.

**Data and Statistical analysis**

The LD_50_ was calculated using the Probit-log analysis. The results are expressed as means±SEM of the percentage values of the test groups relative to the control. The statistical analysis for the animal experiments was carried out using one-way ANOVA followed by Dunnet’s multiple comparisons or chi-square test as appropriate. The results were compared with the control group. A value of *P* less than 0.05 were considered statistically significant.

## Results

**Acute oral toxicity**

There were no visible signs of toxicity and death in animals treated with EOML at doses of 10 and 100 mg/kg for 14 days. The effects of acute oral treatment with EOML are summarized in Table 1. The animals that received various doses of EOML did not exhibit a significant decrease in food and water consumption in 14 days following the treatment (data not shown).

Noticeable signs of toxicity were observed when EOML was administered orally at doses greater than 100 mg/kg. The toxicity signs included tremor, convulsion, abnormal gait and ataxia, increased respiration, decreased activity, unresponsiveness to writhing test, and flaccid paralysis that led to recumbencey (Table. 1). 

The hypo-activity, ataxia, and hyperventilation were seen immediately after administration while the effects of disorientation, convulsion, and syncope were recorded later. The acute toxicity as well as mortality increased progressively in a dose-dependent manner. There was no significant post-treatment drop in body weight in all groups. Visual inspection on necropsy did not reveal any signs of damage to organs. According to the lork’s method, the LD_50_ was 470 mg/kg after oral route dosing in adult rat.

**Intestinal transit time**

**Fig 1:**
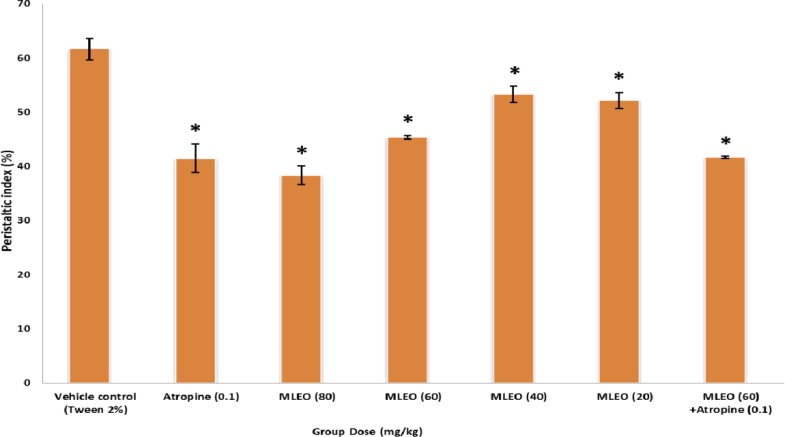
Effect of Menthe longifolia essential oil (MLEO) and atropine on the upper gastrointestinal transit of charcoal meal in normal rat. Results are expressed as mean±SEM; n=6 in each group. Data were analyzed by one way ANOVA followed by Dunnet’s multiple comparisons test. *p<0.05 when compared to control group.

 In the treated groups, EOML (20-80 mg/kg, p.o.) dose-dependently and significantly (*P*<0.05) decreased the normal intestinal propulsive movement and transit of charcoal meal through the small intestine. Atropine (0.1 mg/kg, p.o.) as a standard antispasmodic drug produced greater antimotility effect than the lower dose of EOML at doses of 20-40 mg/kg p.o. EOML at 60 mg/kg showed inhibitory effect (32.43%) equal with atropine group (Figure 1). 

**Table 1:** Acute oral toxicity of *Menthe longifolia *essential oil in rats

**Treatment (mg/kg)**	**D/T**	**Effects**	
		**Mortality latency (h)**	**Symptoms of toxicity **
10	0/3	-	None
100	0/3	-	None
140	0/3	-	Ataxia, hypoactivity, labored respiration, recovery after 10±8 min.
225	0/3	-	Hypoactivity, ataxia, labored respiration, head tics, body tremor, flaccid paralysis, recumbencey, recovery after 58±9 min.
370	1/3	>24, <28	Ataxia, hypoactivity, labored respiration, head tics, body tremor, convulsion, flaccid paralysis, recumbencey, unresponsive to writhing reflex test, recovery after 97±5 min.
600	3/3	>22, <28	Ataxia, hypoactivity, labored respiration, body and head tremor, convulsion, paddling, flaccid paralysis, recumbencey, unresponsive to writhing reflex test
1000	3/3	>1, <3	Ataxia, hypoactivity, labored respiration, body and head tremor, convulsion, paddling, flaccid paralysis, recumbencey, unresponsive to writhing reflex test.

The *Menthe longifolia *essential oil, dissolved in Tween 80 (2%) was administered as single oral doses to 6 groups of 3 rats. All animals were carefully examined for adverse effects (behavioural changes and mortality) for 14 days. Symptoms of toxicity are described for a group; D/T: dead/treated rats; latency: time to death after the dose; (-) none: no toxic symptom during the observation period.

Atropine sulphate produced significant antimotility effect lesser than when the highest dose of EOML (80 mg/kg, p.o.) was used.

**Castor oil–induced diarrhea**

One hundred and thirty minutes after oral administration of castor oil, all of the rats in the control group produced copious and watery diarrhea (Table 2). Pretreatment of the rats with EOML (20 and 80 mg/kg, p.o.) significantly delayed the onset of diarrhea (*p*<0.05). High dose of EOML and loperamide (3 mg/kg, p.o.) showed equal and significant inhibitory effect against diarrhea for 4 hr. In general, the antidiarrheal activity of EOML was significant (*p*<0.05).

Pretreatment with EOML significantly (*p*<0.05) reduced the frequency of diarrhea, defecation and the wetness of the fecal droppings (reduction in the number of wet stools and total stools), and decreased the weight of wet stools and the general diarrhea score, including the hard, mild, and copious stools (Table 3). The standard antidiarrheal drug, loperamide (3 mg/kg, p.o.), and the highest dose of EOML (80 mg/kg, p.o.) produced a more significant (*p*<0.001) inhibitory effect on all of the diarrheal parameters compared with control (Table 3).

**Table 2:** Preventive and curative effect of *Menthe longifolia *essential oil (MLEO) on diarrhea onset and the time course of diarrhea induced by castor oil in rats

**Treatment**	**Dose (mg/kg)**	**Diarrhea onset (min)**	**n**	**Animals with diarrhea (%)**			
				1 h	2 h	3 h	4 h
Control	-	103.66±20.73	6	0	100	100	100
MLEO	80	>240 *	6	0	0 a	0 a	0 a
MLEO	20	170.33±7.33 *	6	0	0 a	50 a	0 a
Loperamide	3	>240 *	6	0	0 a	0 a	0 a

Diarrhea was induced by giving castor oil (2 mL/rat, p.o.).The incidence of diarrhea was checked every hour for 4 h after the castoroil administration. MLEO and Loperamide were given orally 60 min beforethe castor oil treatment. Values are expressed as Mean±S.E.M and the percentage of animal with diarrhea.

* *p*< 0.05 vs vehicle control (ANOVA followed by Dunnett test)

a *p*< 0.05 vs vehicle control (Chi-square test).

**Table 3:** Antidiarrheal activity of *Menthe longifolia *essential oil (MLEO) on the diarrhea parameters that induced by castor oil in rats

**Treatment (mg/kg)**	**Time course of diarrhea**											
	**1 h**			**2 h**			**3 h**			**4 h**		
	F.NO*	F.W*	D.S*	F.NO	F.W	D.S	F.NO	F.W	D.S	F.NO	F.W	D.S
Vehicle control (Tween 2%)	0.00	0.00	0.00	4.00±1.45	3.55±0.93	7.33±1.72	1.66±0.21	1.28±0.08	3.33±0.81	2.33±0.21	1.28±0.09	4.33±1.50
MLEO (80)	0.00	0.00	0.00	0.00 a	0.00 a	0.00 a	0.00 a	0.00 a	0.00 a	0.00 a	0.00 a	0.00 a
MLEO (20)	0.00	0.00	0.00	0.00 a	0.00 a	0.00 a	3.33±1.69	3.20±1.08	5.83±2.31	0.00 a	0.00 a	0.00 a
Loperamide (3)	0.00	0.00	0.00	0.00 a	0.00 a	0.00 a	0.00 a	0.00 a	0.00 a	0.00 a	0.00	0.00 a

The following parameters were recorded at hourly intervals for a period of 4 hr, including:

Values are expressed as Mean±SEM (n=6).

* Fecal numbers (F.NO), fecal weight (F.W), diarrhea score (D.S).

a *p*<0.05 vs. vehicle control.

**Table 4:** Effect of *Menthe longifolia *essential oil (MLEO) on castor oil-induced fluid accumulation in rats.

**Treatment **	**Dose (mg/kg)**	**Fluid volume (ml)**	**% inhibition**
Control	-	3.25± 0.68 a	-
MLEO	80	1.03± 0.19^a^,^b^,^c^	68.30
MLEO	20	2.75± 0.56	15.38
Loperamide	3	1.83 ± 0.20^a^,^b^,^c^	43.69

Values are expressed as as Mean±SEM (n=6).

a *p*<0.05,

b *p*<0.01 and

c *p*<0.001vs. vehicle control.

**Castor oil-induced intestinal transit**

In the control group, the charcoal meal moved farther (85.22±4.31) in comparison to the normal intestinal transit test (61.66±1.99). EOML significantly (*p*<0.05) decreased the peristaltic index and transit of charcoal meal through the small intestine in castor oil treated rats and this effect was found to be dose-dependent (data not shown). Significant inhibition at the maximum dose (80 mg/kg) was 30±2.22. 

**Castor oil-induced fluid accumulation**

Oral administration of castor oil produced a significant (p<0.05) increase in the intestinal fluid volume of castor oil-treated groups compared with the control group treated with distilled water (10 mL/kg, p.o.). Compared to the control group, pretreatment of the animals with EOML (80 mg/kg, p.o.) significantly (p<0.05–0.01) inhibited castor oil-induced fluid accumulation in rats (Table 4). 

Loperamide (3 mg/kg) produced a greater (p<0.001) inhibitory effect on fluid accumulation test. Loperamide and the highest dose of EOML (80 mg/kg) had similar activity. The intestinal fluid of the animals pretreated with EOML and loperamide were more viscous than those of the distilled water-treated rats.

## Discussion

In the current study, antidiarrheal properties and acute toxicity of the essential oil of *M. longifolia* were investigated. Three animal models were used which widely applied because of their simplicity and reproducibility. The present study revealed that EOML possesses antidiarrheal activity in all of the used. Oral medication with EOML may be life-threatening in higher doses. Previously, we showed that EOML has a relaxant effect on rat ileum smooth muscle (Jalilzadeh-Amin et al., 2012 ▶) as confirmed here with *in vivo* tests. 

Oral administrations of graded doses of EOML in rats gave an LD_50_ value of 470 mg/kg. Based on the classification of Pascoe (1983), EOML was found to be very toxic to rats when given via the oral route and moderately toxic via oral according to the classification of Loomis and Hayes (1996). The essential oil obtained from a Spanish collection of *M. longifolia* had an LD_50_ of 437, 4 mg/kg on the mouse (Perez Raya et al., 1990 ▶), but LD_50_ information relating to this herb was not reported by others (Odeyemi et al., 2009 ▶). 

 Odeyemi et al., (2009) ▶ stated that the essential oil from *M. longifolia* may not be completely safe. Likely, these preliminary data suggested that EOML was practically a toxic agent, because some of the rats died at high doses of EOML.

The main constituents of the essential oil from *M. longifolia* leaves obtained from the Urmia, included 1,8-cineole (18.93%), pulegone (15.55%) and isomenthone (11.80%) (jalilzadeh-Amin et al., 2011 ▶). Menthone has been reported to be a growth inhibitor, (Slama, 1987 ▶) whereas pulegone, a potent abortifacient, is metabolized to a series of hepatotoxins that causes liver cancer (Nelson, 1995 ▶). Additionally, it has been shown that pulegone can rapidly destroy liver (Thomassen et al., 1990 ▶) and inhibit the cytochrome P_450 _(Moorthy, 1991 ▶). The treatment doses utilized in the present study were safe and demonstrated no untoward behavioral effects in the rats. 

Castor oil-induce inflammation in the intestinal mucosa led to permeability changes in the intestinal mucosal membranes to water and electrolytes which were associated with the release of endogenous substances such as prostaglandins and nitric oxide (Franca et al., 2008) which resulted in the increase of the volume of intestinal watery content. The balanced absorption and secretion in the ileum were controlled through sympathetic nervous by means of mucosal adrenoceptors (Berthelsen and Pettinger, 1977 ▶). The anti-motility effect of EOML appears to be due to mechanisms independent of activation of cholinergic receptors, since atropine, a cholinergic antagonist, failed to reverse its action in the gastrointestinal transit test. Other mechanisms such as adrenergic and/or nonadrenergic-noncholinergic (NANC) systems are also suspected.

Pulegone and 1,8-cineole were the principal chemical components of EOML which have been identified in our previous study (Jalilzadeh-Amin et al., 2012 ▶). The presence of such terpenes in the essence could be attributed to the antidiarrheal activity of EOML. These naturally occurring terpenes were able to reduce the normal and altered propulsive movement induced by castor oil (Jalilzadeh-Amin and Maham, 2013 ▶; Jalilzadeh-Amin and Maham, In press). It is well known that terpenoides can act as antispasmodic agents by involving calcium antagonism (Shabana et al., 2005 ▶). It has been demonstrated that pulegone inhibits contractions induced by spasmogens in smooth muscle isolated from rat and guinea-pig intestine (De Urbina et al., 1990 ▶; Brankovic et al., 2009 ▶) and myometrium (Soares et al., 2005 ▶). Interestingly, cineole provided an antispasmodic activity on the isolated ileum (Magalhaes et al., 1998 ▶). In addition, there is probably an involvement of cineole, which has been shown to block nerve excitability (Leal-Cardoso et al., 2004 ▶) and relax isolated smooth muscle (Coelho-de-Souza et al., 1997 ▶). Recently, it has been reported that *M. longifolia* have calcium channel blocking activity (Shah et al., 2010 ▶). This mechanism can be an acceptable explanation to its spasmolytic effect.

EOML produced a significant reduction in the severity of diarrhea and gastrointestinal transit. It is well known that drugs affecting motility, frequency, and consistency of diarrhea also affect secretion (Hsu, 1982 ▶). EOML dose-dependently inhibits the altered intestinal transit and intraluminal fluid accumulation as well as loperamide. Therefore, in clinically occurred diarrhea that results from impaired intestinal functions and motility (Gurgel et al., 2001 ▶) it might be an option. 

EOML inhibited the volume of intestinal fluid accumulation in a dose-related manner suggest that EOML may enhance electrolyte reabsorption consistent with the inhibition of hyper-secretion or may encourage the absorption of other intestinal contents. The precise mechanism of hyper-secretion affected by EOML is not clear, likely delay in intestinal motility causes further absorption of water from faeces and may additionally contribute to reducing its watery texture. 

The *in vivo* effects of EOML in the present study are consistent with our previous findings, which indicated a reduction of total faeces weight and soft faeces frequency by pulegone (Jalilzadeh-Amin and Maham, 2013 ▶) and 1,8-cineole (Jalilzadeh-Amin and Maham, In press). 

Anti-inflammatory activity of pulegone and cineole through the inhibition of inflammatory mediators releasing were demonstrated previously (Kawata et al., 2008 ▶; Santos and Rao, 2000 ▶). In fact, these terpens blockade prostaglandin and other inflammatory mediator’s formation in diarrhea which may in part explain its anti-secretory effect. Finally, it is interesting to highlight that α-pinene, another monoterpenes of the essential oil of *M. longifolia*, could also enhance the antidiarrheal activity of EOML, since it has shown anti-inflammatory effects in animal models (Guimaraes et al., 2013 ▶). Other EOML constituents also appear to be involved in its antidiarrheal action. Further studies are needed to clarify the mechanism of action and the components responsible for these pharmacological effects.

In conclusion, EOML possesses antidiarrheal activity due to inhibitory effects on both gastrointestinal propulsion and fluid secretion. These data give a scientific base justifying the folkloric use of EOML. *M. longifolia* usage as a vegetable and an herbal medicine should be accompanied with cautious because it may have serious side effects.
